# Metabolome and microbiome of chronic periapical periodontitis in permanent anterior teeth: a pilot study

**DOI:** 10.1186/s12903-021-01972-8

**Published:** 2021-11-23

**Authors:** Yun Huang, Peng Zhou, Siqi Liu, Wei Duan, Qinqin Zhang, Ying Lu, Xin Wei

**Affiliations:** 1 Jiangsu Province Key Laboratory of Oral Diseases, Department of Conservative Dentistry and Endodontics, Stomatological Hospital, Nanjing Medical University, Nanjing, China; 2 Department of Operative Dentistry and Endodontics, Affiliated Stomatological Hospital of Nanjing Medical University, Nanjing, Jiangsu China; 3 Jiangsu Province Engineering Research Center of Stomatological Translational Medicine, Nanjing, China

**Keywords:** Periapical periodontitis, Inflammatory grades, Metabolome, Microbiome

## Abstract

**Background:**

Periapical periodontitis is a common oral inflammatory disease that affects periapical tissues and is caused by bacteria in the root canal system. The relationship among the local metabolome, the inflammatory grade, and the type and abundance of microorganisms associated with periapical periodontitis is discussed in this study.

**Methods:**

The inflammatory grades of periapical samples from 47 patients with chronic periapical periodontitis in permanent anterior teeth were determined based on the immune cell densities in tissues subjected to haematoxylin and eosin staining. The metabolome was evaluated using ultrahigh-performance liquid chromatography-quadrupole time-of-flight mass spectrometry, followed by principal component analysis and orthogonal partial least squares discriminant analysis. The microbiome was accessed using 16 S rRNA high-throughput sequencing. The differences in the metabolomes and microbiomes of the periapical periodontitis samples were assessed using Spearman’s correlation analysis.

**Result:**

*N*-acetyl-D-glucosamine, L-tryptophan, L-phenylalanine, and 15 other metabolites were identified by the comparison between samples with severe inflammation and mild or moderate inflammation. Four amino acid metabolism pathways and one sugar metabolism pathway were associated with the inflammatory grade of periapical periodontitis. The abundance of *Actinomycetes* was negatively correlated with the abundance of glucosamine (GlcN), while the abundance of *Tannerella* was positively correlated with the abundance of L-methionine.

**Conclusions:**

The local metabolome of periapical periodontitis is correlated with the inflammatory grade. The abundance of the local metabolites GlcN and L-methionine is correlated with the abundance of the major microorganisms *Actinomycetes* and *Tannerella*, respectively.

## Background

Periapical periodontitis is a common oral inflammatory disease that affects periapical tissues and is mainly caused by bacteria inside the root canal system; periapical periodontitis leads to periodontal ligament destruction, alveolar bone resorption, and granulation tissue formation [1]. When the alveolar bone is heavily damaged and absorbed during periapical periodontitis and inflammation cannot be cured by root canal therapy alone, surgical resection is needed [2]. For cases that are difficult to cure with periapical surgery, it is necessary to remove the affected teeth and eliminate the source of infection in order to return the condition of the patient to normal. In addition, the infectious bacteria and virulence factors associated with some clinically severe periapical diseases can lead to subacute infected endocarditis or even septicemia in immunodeficient or immunocompromised patients and transplant patients, and these consequences can be life-threatening [3]. Due to the severe prognosis of periapical periodontitis, the mechanisms underlying the development of this disease need to be further studied.

Previous studies have indicated that there are many kinds of microorganisms in periapical periodontitis [4], and immune cells accumulate around the periapical tissue and secrete proinflammatory cytokines after tissue infection or injury [5]. Lymphocytes secrete proinflammatory cytokines, such as tumour necrosis factor (TNF), interleukin-1 (IL-1) and interleukin-6 (IL-6), and these cytokines can directly or indirectly stimulate the activity of osteoclasts and result in alveolar bone absorption, suggesting a possible role of immune cells in alveolar bone absorption [4].

Metabolism is a general term for chemical reactions that sustain life, and it mainly includes glucose metabolism, lipid metabolism, and amino acid metabolism. Microorganisms and local immune alterations affect local metabolism, and local metabolic changes might in turn affect inflammatory grades. For example, tryptophan is broken down into kynurenine under the action of indoleamine 2,3-dioxidase (IDO) in chronic nephritis, and the activity of IDO is positively correlated with the serum level of kynurenine and the severity of chronic nephritis [6]. Interactions between metabolism and inflammatory factors have been described, and inflammation could cause corresponding metabolic changes [7]. Metabolomics is a new approach that allows the simultaneous qualitative and quantitative analysis of all low-molecular-weight metabolites (< 1 kDa) in biological samples that are produced during an inflammatory or specific physiological period [8, 9]. Previous studies revealed unique metabolic changes in patients with primary sclerosing cholangitis through the metabolomics analysis of portal venous and bile, providing a potential target for the development of new therapies [9]. The possible role of local metabolic changes caused by host immune/inflammatory alterations in response to periapical periodontitis needs to be discussed.

Periapical periodontitis is an oral inflammatory disease that affects periapical tissues and is caused by bacteria; however, little information is available in the scientific literature about the correlation between the local microbiome and the levels of inflammation associated with periapical periodontitis. Here, we investigated the relationship among the local metabolic differences, the inflammatory grade, and the type and abundance of microorganisms associated with periapical periodontitis. This study will provide potential targets for new strategies to prevent the development of and resolve periapical periodontitis.

## Materials and methods

### Patient selection

In this study, 47 patients (age 18 to 45 years) with chronic periapical periodontitis in anterior teeth after root canal therapy were enrolled from March 2018 to January 2020 from the Department of Endodontics of Jiangsu Stomatological Hospital (Nanjing, China). The diagnosis of chronic periapical lesions was confirmed by two endodontic specialists, and all the patients provided written informed consent. Patients who presented with systemic diseases, such as hypertension and diabetes, or recently had a history of antibiotic use were excluded from the study.

### Sample procedure

Periapical tissues were collected during the process of periapical microsurgery, immediately washed and stored in sterile phosphate-buffered saline. Each periapical tissue sample was divided into 3 parts. One part of the tissue was incubated in 10% formalin solution for histopathological analysis. One part of the tissue was frozen in liquid nitrogen within 45 min and stored at -80 ℃ for metabolome analysis. One part of the tissue was frozen in liquid nitrogen within 45 min and stored at -80 ℃ for microbiome analysis.

### Histopathological analysis

#### Haematoxylin and eosin (H&E) staining

The periapical specimens were fixed in 10% formalin buffer for 72 h. Then, they were washed in running water for 2 h, dehydrated in ascending dilutions of ethanol (70% ethanol, 95% ethanol for 30 min, anhydrous ethanol I and anhydrous ethanol II for 40 min), diazotized in xylene (xylene I, xylene II for 20 min, and xylene III for 40 min), and finally embedded in paraffin. Paraffin Sect. (5 μm thick) were heat immobilized, deparaffinized with xylene, and rehydrated using a graded series of ethanol. H&E staining was performed, and then, the periapical tissues were observed with an optical microscope at 200 times magnification (Leica, Germany).

#### Inflammatory grade

The degree of inflammation in periapical periodontitis was divided into 3 levels based on the densities of immune cells in the tissues as observed under an optical microscope: grade I, inflammatory cells accounted for less than 1/3 of each field; grade II, inflammatory cells accounted for 1/3 to 2/3 of each field; and grade III, inflammatory cells accounted for more than 2/3 of each field [10].

### Metabolome analysis

#### Metabolite extraction

A 30-mg piece of periapical specimen collected from microapical surgical resection was placed in a 1.5-mL Eppendorf tube with 600 µL methanol (2:2:1 v/v/v; 2 µg/mL 2-chloro-L-phenylalanine as the internal standard) and then vortexed for 30 s. Porcelain beads were added to the mixture, and a mixed grinding apparatus (JXFSTPRP-24, Shanghai, China) was used to homogenize the sample (45 Hz, 4 min). Subsequently, ultrasonic treatment in an ice water bath was performed for 5 min and repeated 3 times. The sample was incubated at − 20 ℃ for 1 h. Then, the mixture was centrifuged at 12,000 rpm at 4 ℃ for 15 min. Then, 200 µL supernatant was transferred to a new Eppendorf tube for vacuum drying, mixed with 200 µL extract (acetonitrile water volume ratio: 1:1), vortexed for 30 s, and subjected to ultrasonic treatment in an ice water bath for 10 min. Next, each sample was centrifuged at 12,000 rpm at 4 ℃ for 15 min. After centrifugation, an aliquot of 75 µL supernatant was transferred to a sample vial (2 mL) for UHPLC/QTOF–MS (LC–MS) analysis. The quality control (QC) sample was prepared by mixing equal amounts of 10 µL from each sample, which together represented a QC standard for all the samples of periapical periodontitis. Then, 75 µL of each mixture was used for testing. To assess the repeatability of the analysis, the QC sample was injected once after every five samples tested during acquisition [11].

#### Metabolomic profiling technology

LC–MS analysis of periapical specimens was carried out on an Agilent 1290 Infinity UHPLC system (Agilent, California, America) in positive mode and negative mode [12]. The chromatographic column was a UPLCBEHAmide column (1.7 mm, 2.1*100 mm, Waters, Milford, MA, USA). The optimal mobile phase was composed of “A” (25 mM ammonium acetate and 25 mM ammonium hydroxide in water (pH 9.75)) and “B” (acetonitrile). The gradient elution of the samples was performed as follows: 0–0.5 min, 5% B; 7 min, 65% B; 8–9 min, 40% B; 9.1–12 min, 95% B. The flow rate was set to 0.5 mL/min. The injection volume of the test sample was 1 µL.

The mass spectrometer was operated using TripleTOF6600 triple four-pole time-of-flight mass spectrometry (ABSciex, America). Collision energy (CE) was 30 eV. The parameters of the electron spray ionization (ESI) source were set as follows: ion source gas 1, 60 psi; ion source gas 2, 60 psi; source temperature, 600 ℃; and ion spray voltage floating (ISVF), 5 kV or − 4 kV in positive or negative mode, respectively.

#### Data processing and multivariate data analysis

The mass spectrometer original data from the LC–MS analysis of the metabolites were converted to the common (mzXML) format by ProteoWizard. Then, the converted data were imported into XCMS 3.2 software (http://bioconductor.org/packages/release/bioc/html/xcms.html) to generate a data matrix that involved the normalized peak intensity, exact mass, and retention time. These metabolome data were input into the metaboanalyst 4.0 platform (https://www.metaboanalyst.ca) for multivariate statistical analysis. Principal component analysis (PCA) and a partial least squares-discriminant analysis (PLS-DA) model constructed by the pattern recognition method were used to identify the underlying difference in the metabolites between the groups with different inflammatory grades.

### Microbiome analysis

#### Microorganism extraction

A 30-mg piece of periapical specimen in an Eppendorf tube was treated for total genomic DNA extraction according to the manufacturer’s instructions (E.Z.N.A.® Stool DNA Kit, Omega Bio-tek, Norcross, GA, U.S.). The yield and purity of the DNA were measured using a Nanodrop 8000 (Thermo Scientific, Wilmington, DE, USA). DNA was used to study microbial diversity by amplifying the V3–V4 region of the 16 S rRNA gene using the universal primers 338 F (5′-GTACTCCTACGGGAGGCAGCA-3′) and 806R (5′-GTGGACTACHVGGGTWTCTAAT-3′). The PCR mixture contained 30 ng template DNA, 1 µL upstream primer (5 µmol/L), 1 µL downstream primer (5 µmol/L), 1 µL BSA (1 ng/µL), and 12.5 µL 2×Taq Plus Master Mix, and sterile Milli-Q water was used to adjust the final volume to 25 µL. PCR was performed with an initial denaturation at 94 ℃ for 5 min, 45 cycles of denaturation at 94 ℃ for 30 s, annealing at 50 ℃ for 30 s, and extension at 72 ℃ for 60 s, and a final extension at 72 ℃ for 10 min. Post-amplification quality control was performed by electrophoresis with a 2% agarose gel. PCR products were purified with AMPure XP Beads (Beckman Coulter, Milan, Italy).

#### Data analysis

The microbiome data acquired from the 16 S rRNA high-throughput sequencing were imported and analysed by QIIME 1.8.0 software (http://qiime.org/). OTUs (operational taxonomic units) were picked at 99% similarity with UCLUST clustering methods to predict the abundances of species based on OTU data according to the Silva databases.

## Statistical analysis

The metabolites with a variable importance in project (VIP) > 1 in the PLS-DA model and a *p* value *<* 0.05 in the analysis of variance were considered potential biomarkers identified by the LC–MS analysis. The affected metabolic pathways were explored via Metabolomic Pathway Analysis software (https://www.metaboanalyst.ca), and those with an impact value > 0.1 and a *p* value < 0.05 were selected as the significantly different pathways. Analysis of variance was conducted to compare the relative abundance of major microorganisms associated with periapical periodontitis with different inflammation grades, and all the data were analysed with SPSS 17.0 software (Chicago, USA). Significant differences between the metabolome and microbiome of periapical periodontitis were assessed using Spearman’s correlation analysis. The R language (pheatmap package) was used to analyse the Spearman correlation coefficient, and the resulting numerical matrix was displayed using a heat map.

## Results

### Haematoxylin and eosin (H&E) staining and inflammatory grade

**Fig. 1:**
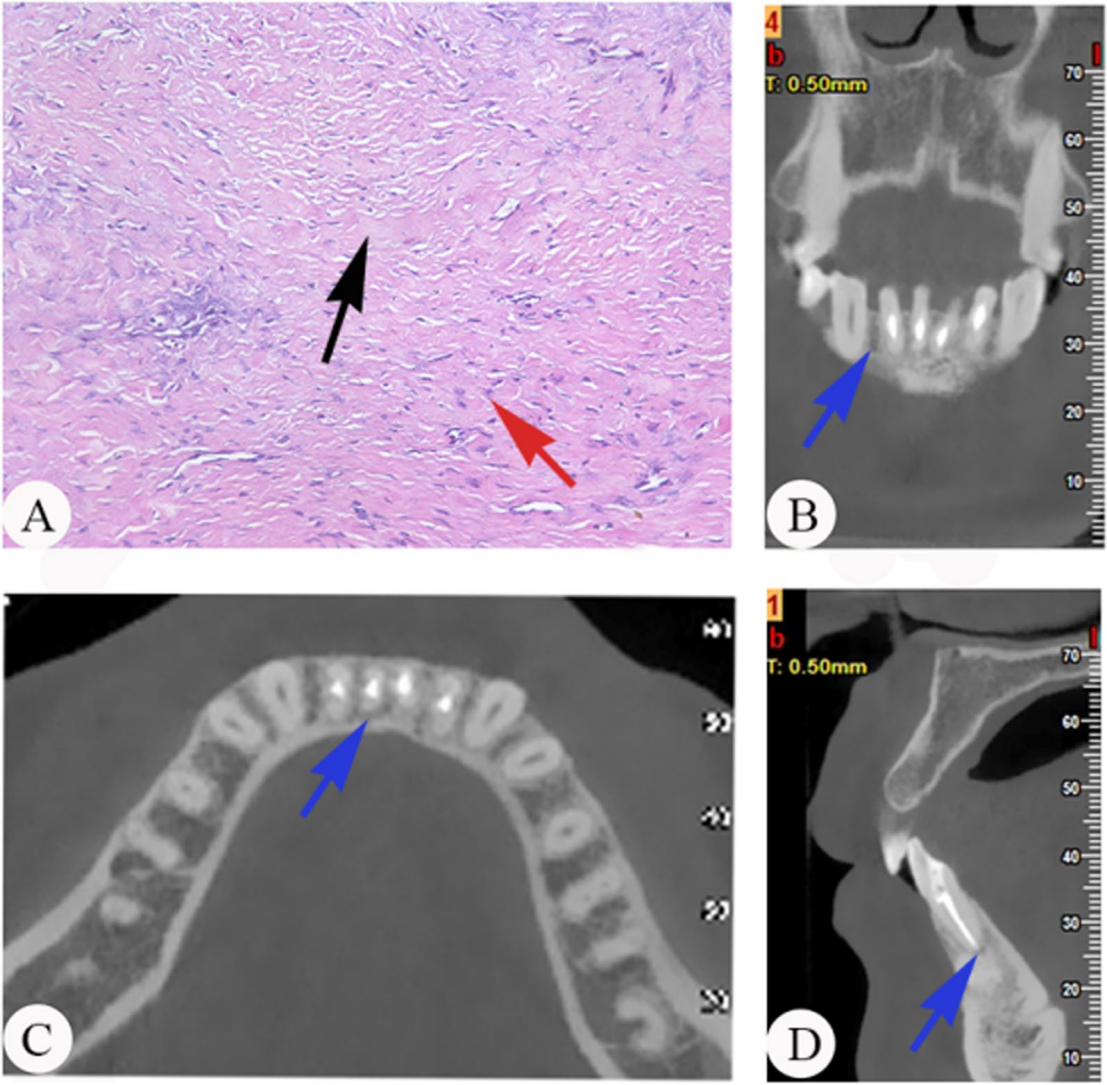
Examples of included cases with histopathology and CBCT sections. **A** Histopathology of periapical lesions (× 200, H&E staining) in grade I. Fibrous/scar tissue with slight inflammation. The immune cells (red arrow) and proliferative fibrous tissue (black arrow) exist in the periapical tissue. **B** CBCT, coronal section of periapical tissue, blue arrow points at periapical lesions. **C** CBCT, horizontal section of periapical tissue, blue arrow points at periapical lesions. **D** CBCT, sagittal section of periapical tissue, blue arrow points at periapical lesions

**Fig. 2:**
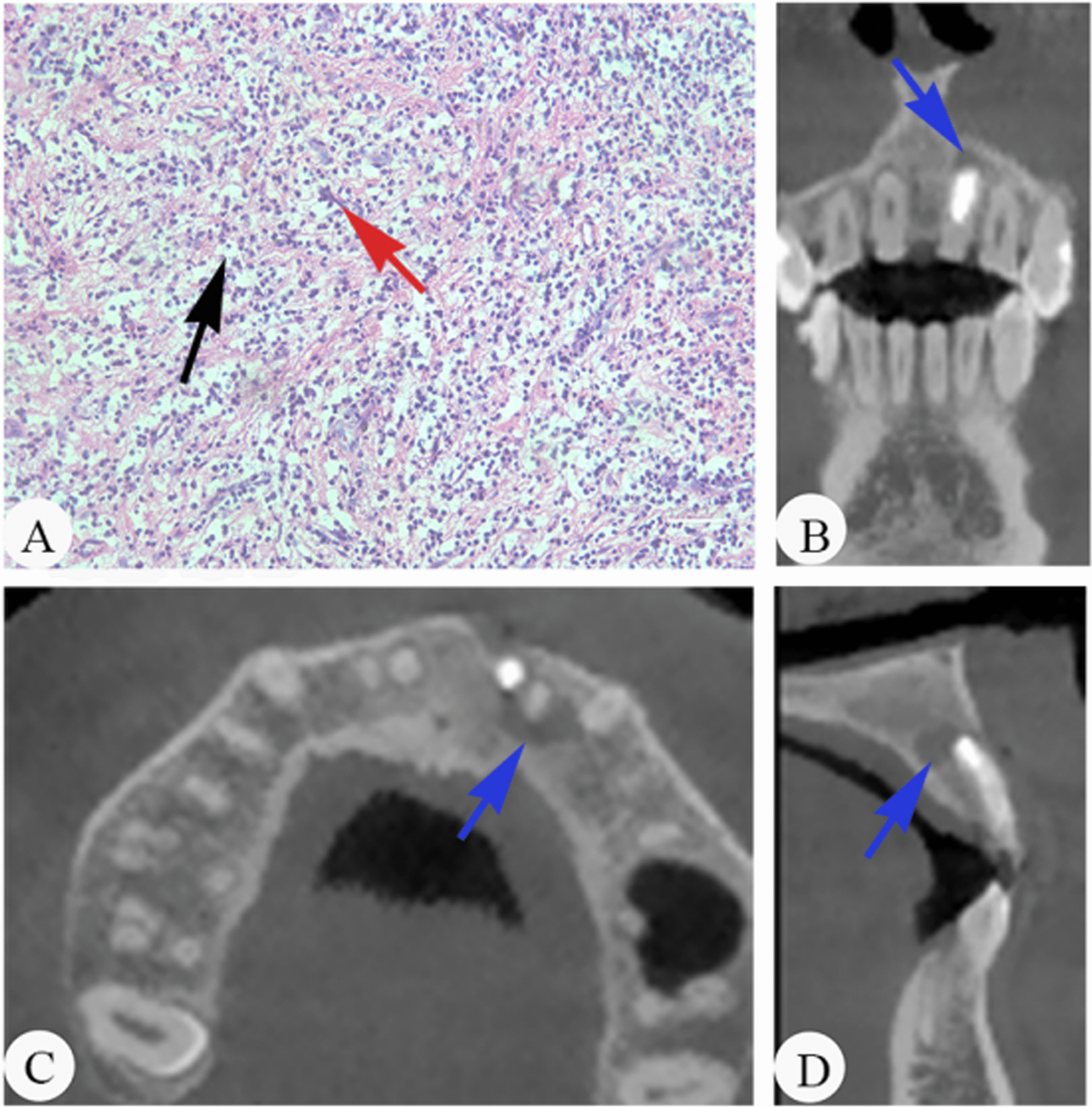
Examples of included cases with histopathology and CBCT sections. **A** Histopathology of periapical lesions (× 200, H&E staining) in grade II. Fibrous/scar tissue with moderate inflammation. The immune cells (red arrow) and proliferative fibrous tissue (black arrow) exist in the periapical tissue. **B** CBCT, coronal section of periapical tissue, blue arrow points at periapical lesions. **C** CBCT, horizontal section of periapical tissue, blue arrow points at periapical lesions. **D** CBCT, sagittal section of periapical tissue, blue arrow points at periapical lesions

**Fig. 3:**
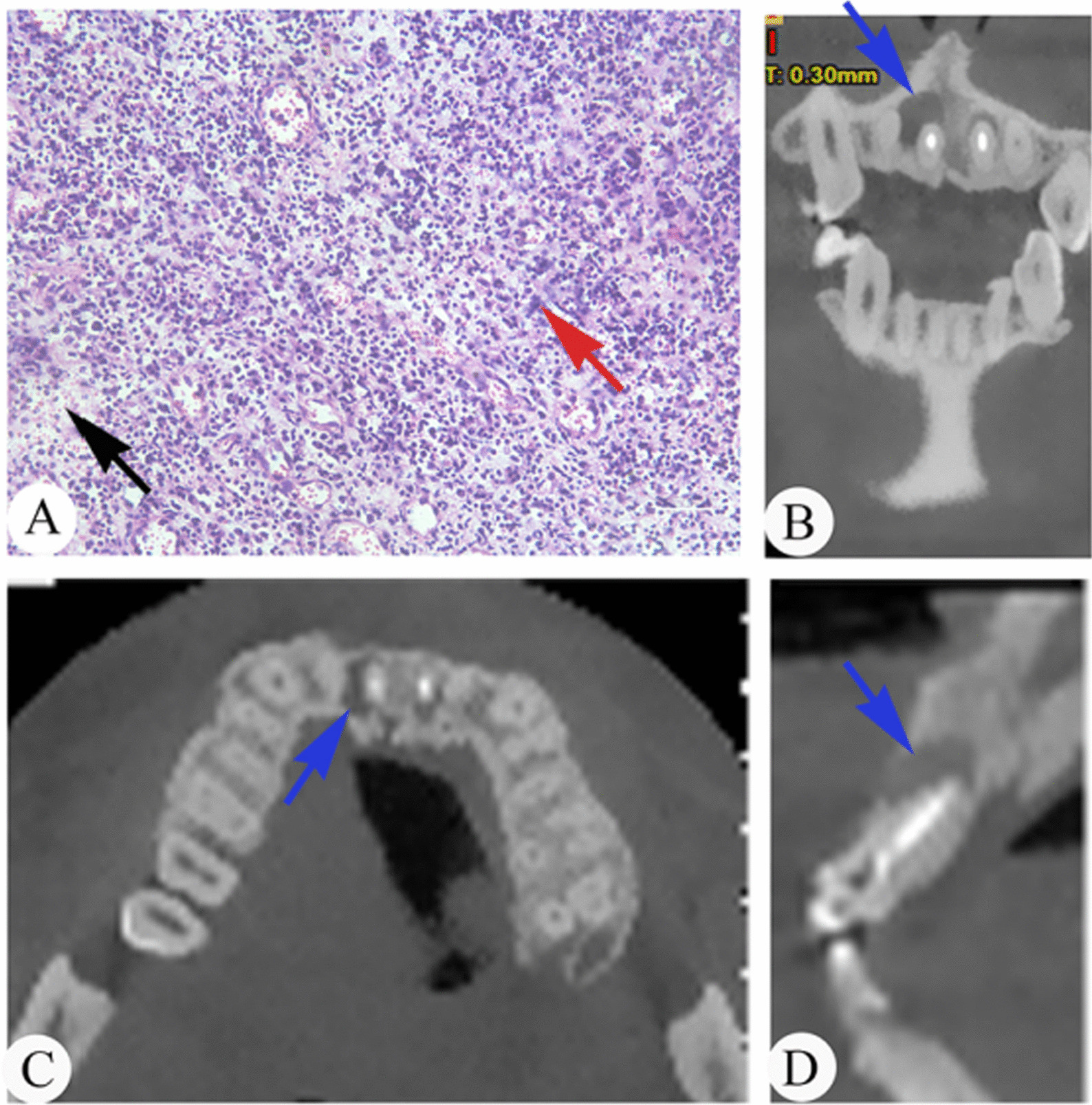
Examples of included cases with histopathology and CBCT sections. **A** Histopathology of periapical lesions (× 200, H&E staining) in grade III. Fibrous/scar tissue with severe inflammation. The immune cells (red arrow) and proliferative fibrous tissue (black arrow) exist in the periapical tissue. **B** CBCT, coronal section of periapical tissue, blue arrow points at periapical lesions. **C** CBCT, horizontal section of periapical tissue, blue arrow points at periapical lesions. **D** CBCT, sagittal section of periapical tissue, blue arrow points at periapical lesions.

Among 47 specimens, the histopathological examination showed 11 specimens (23.4%) with slight inflammation (grade I), 20 specimens (42.6%) with moderate inflammation (grade II), and 16 specimens (34.0%) with severe inflammation (grade III). The lesions of periapical periodontitis in the sections stained with haematoxylin and eosin (H&E) included immune cells (dominantly macrophages and lymphocytes), proliferative capillaries, and fibrous tissue (Figs. 1A, 2A, 3A). After light microscopic examination, the lesions of the periapical tissues were divided into three grades based on inflammatory cell infiltration. Then, the samples analysed to determine the relationship of the local metabolome and the type and abundance of microorganisms were subsequently divided into three groups based on the different inflammatory grades: grade I, grade II, and grade III (Figs. 1, 2, 3). Figures 1, 2 and 3 show examples of histopathology and CBCT of the periapical periodontitis specimens.

### Metabolome analysis of periapical periodontitis

#### Metabolome analysis

**Fig. 4:**
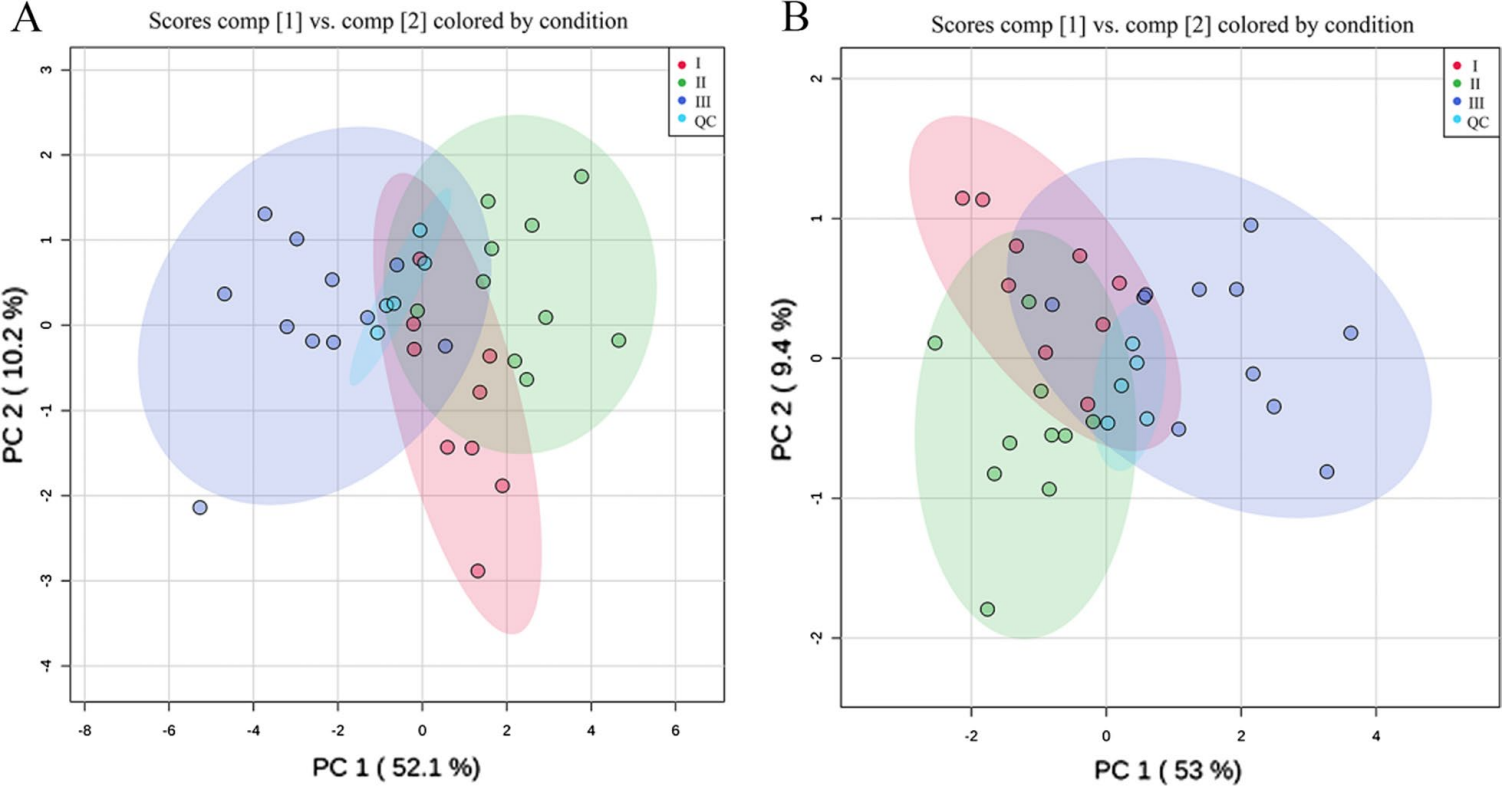
PCA statistical analysis model of periapical periodontitis using UPLC-QTOF/MS. The abscissa represents the first principal component and the ordinate represents the second principal component. **A** Positive ion mode, **B** negative ion mode. The red, green, and purple scatters represent the metabolic profiles of periapical periodontitis as grade I, grade II, and grade III, respectively. Light blue scatters represent the QC samples. Each scatters represents a sample, and the closer the scatter is, the higher the similarity of the principal components of the samples exists

In the PCA score plot (Fig. 4A, B), the QC samples (light blue scatters) were clustered together in the centre of the plot (inside a small light blue circle in Fig. 4A, B, respectively), indicating satisfactory stability regarding sample quality and analytical methods [13]. The samples inside the red, green, and purple circles represent the metabolic profiles of periapical periodontitis with grade I, grade II and grade III, respectively. Moreover, the graph of the PCA statistical analysis model of periapical periodontitis also showed that the periapical periodontitis samples in the same group were clustered in one area (inside the red, green and purple circles of Fig. 4A, B, respectively), meaning that there was similarity in the main component in the samples with the same inflammatory grades (Fig. 4A, B). The metabolic profiles in the graph of the PCA model from of periapical periodontitis with different inflammatory grades were obviously separated and different in both negative and positive ion modes (red, green, and purple circle of Fig. 4A, B), which suggested that metabolic differences existed among three different inflammatory grades.

**Fig. 5:**
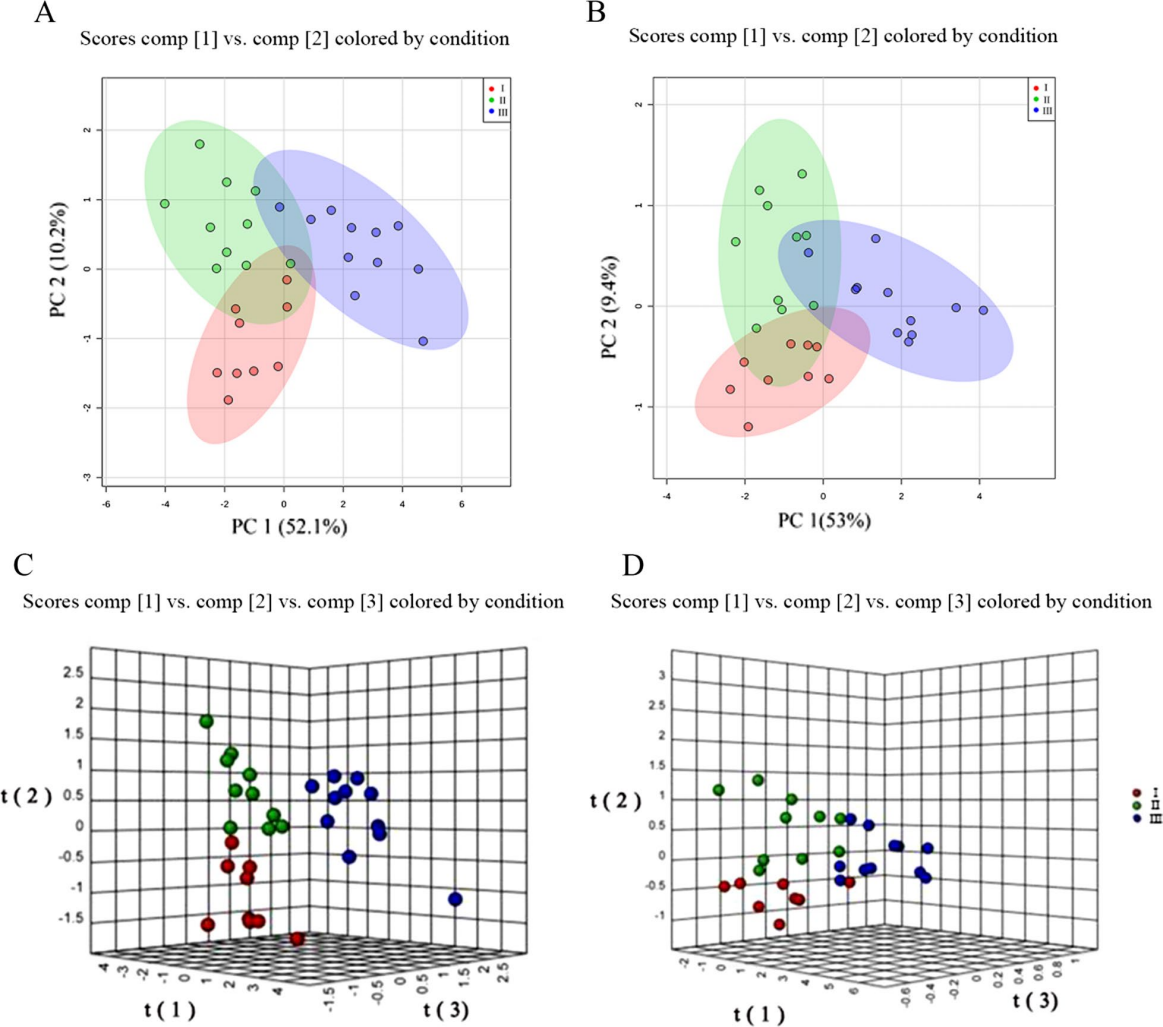
PLS-DA and 3D PLS-DA statistical analysis models of periapical periodontitis. **A** PLS-DA statistical analysis models of periapical periodontitis in positive ion mode; **B** PLS-DA statistical analysis models of periapical periodontitis in negative ion mode; **C** 3D PLS-DA statistical analysis models of periapical periodontitis in positive ion mode; **D** 3D PLS-DA statistical analysis models of periapical periodontitis in negative ion mode. Red, green, and purple spots respectively represent the samples of periapical periodontitis at grade I, grade II, and grade III. Each scatters represents a sample. The closer the scatter is, the smaller of the difference between the samples exists

The PLS-DA score plots (Fig. 5C, D) revealed that there was a clear separation in the different grades of periapical periodontitis, while less separation was observed within any particular grade (Fig. 5A, B). This result indicated that there was a significant intergroup difference among the different inflammatory grades. The development of periapical periodontitis may be associated with important metabolic disorders and pathological physiological changes. Of note, the three different groups of periapical periodontitis partly overlapped (Fig. 5A, B), which suggested that the changes in inflammation were gradual and continuous.

#### Metabolite identification

**Fig. 6:**
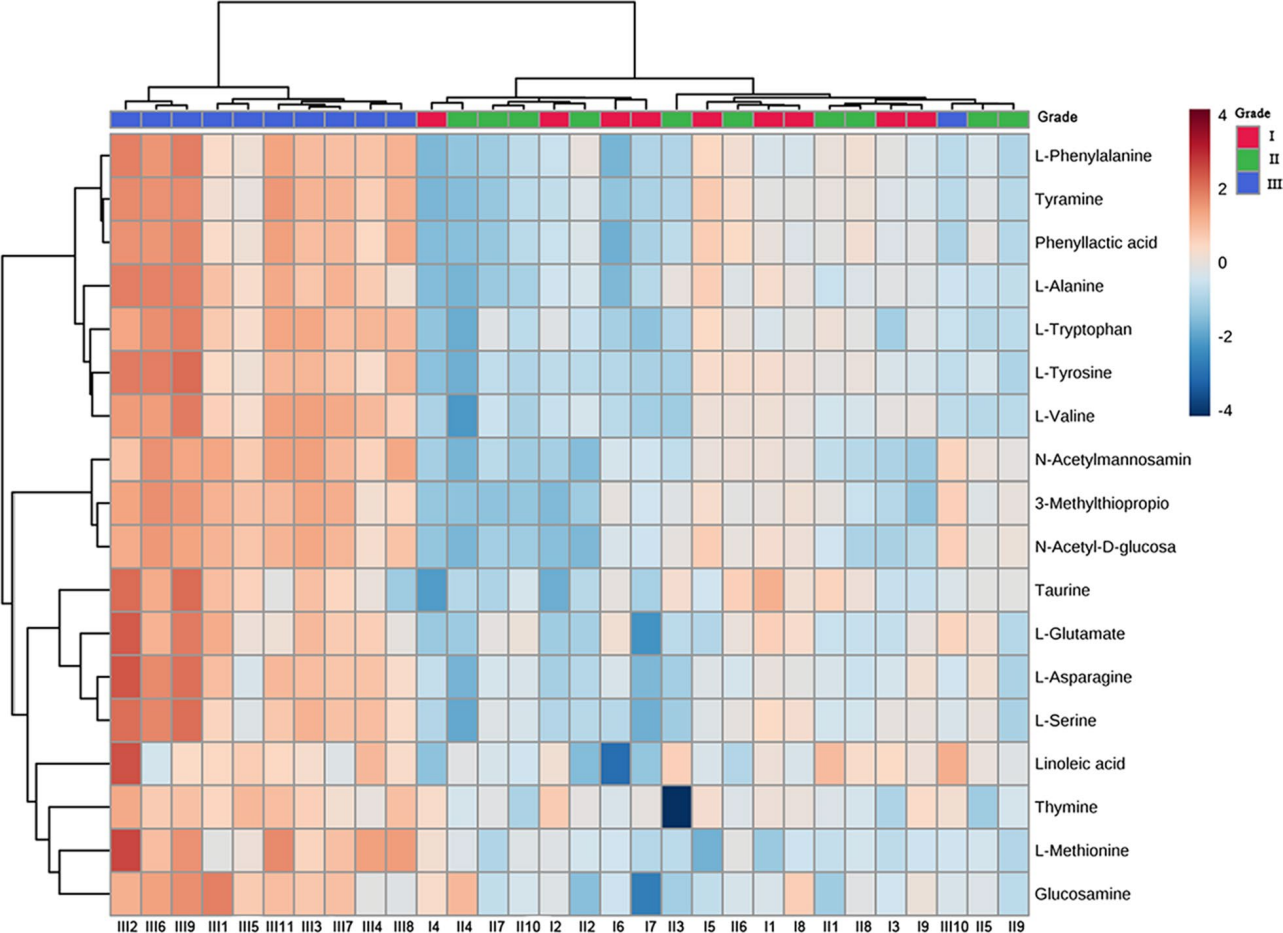
Heat map of differential metabolite abundance levels of periapical periodontitis in different groups. The color saturation in the heat map indicates the expression of metabolites. The rows represent the inflammatory grade of metabolites and the columns show the expression of metabolites. Blue indicates the lowest expression of metabolites and brown indicates the highest expression of metabolites, and white indicates the mean value expression of metabolites

Eight potential biomarkers, such as *N*-acetyl-D-glucosamine, were identified in the three periapical periodontitis groups by LC–MS analysis in cation mode (Table 1), while ten potential biomarkers, such as L-phenylalanine, were obtained in anion mode (Table 2). These differential metabolites identified above were involved in changes in amino acid metabolism, sugar metabolism, and fatty acid metabolism during the progression of periapical periodontitis. The potential and possible roles of metabolites in periapical periodontitis are shown in Tables 1 and 2. Then, a heat map was constructed to visualize the expression of these differential metabolites in the different periapical periodontitis groups (Fig. 6). The heat map showed that the colour of the metabolites in the grade I and II groups only slightly changed, while that in the grade III group obviously changed. This result indicated that the expression of the metabolites associated with periapical periodontitis increased more obviously in the grade III group than in the grade I and II groups.

**Table 1:** Potential bio-markers of periapical lesions in cation mode

Name	VIP	*P* value	Potential role	References
*N*-Acetylmannosamine	2.5741	3.16E−09		
*N*-acetyl-D-glucosamine	2.0183	2.18E−07	Anti-inflammatory, antioxidant	[14]
3-methylthiopropionate	1.9203	1.49E−07	Methionine metabolites	[15]
Tyramine	1.423	6.06E−05		
Benzene lactic acid	1.3575	0.00013	Improving the immune function	[16]
L-Tyrosine	1.2729	3.35E−05		
Thymine	1.2627	2.77E−05		
L-Methionine	1.2441	7.25E−06	Anti-inflammatorry,Inhibiting osteoclast	[15]

**Table 2:** Potential bio-markers of periapical lesions in anion mode

Name	VIP	*P* value	Potential Role	References
L-Phenylalanine	1.7786	6.02E**−**06	Inhibiting 5-phenylalanine decarboxylase	[17]
L-Glutamate	1.5181	0.000137	Increasing IFN-γ and IL-10	[18]
L-Tryptophan	1.5134	3.79E**−**06	Decreasing Il-1β, Il-6, IL-8, TNF-α	[19]
L-Alanine	1.4168	1.44E**−**05	Decreasing TNF-a	[20]
Glucosamine	1.3915	0.000693	anti-inflammary, antioxidant	[14, 19]
L-Serine	1.3393	4.59E**−**05	Regulating of T-cell Proliferation	[21]
L-Asparagine	1.2961	1.93E**−**05	Decreasing TNF-α and IL-1β	[22]
L-Valine	1.1814	2.12E**−**06	Generating immunoglobulin Decreasing TNF-α, IL-6, INF-γ, IL-1β and IL-17	[23]
Linoleic acid	1.1752	0.011785	No influence	[24]
Taurine	1.0241	0.013787	Decreasing TNF-α, IL-6 and Oxidative stress	[25]

#### Metabolomic pathway interpretation

**Fig. 7:**
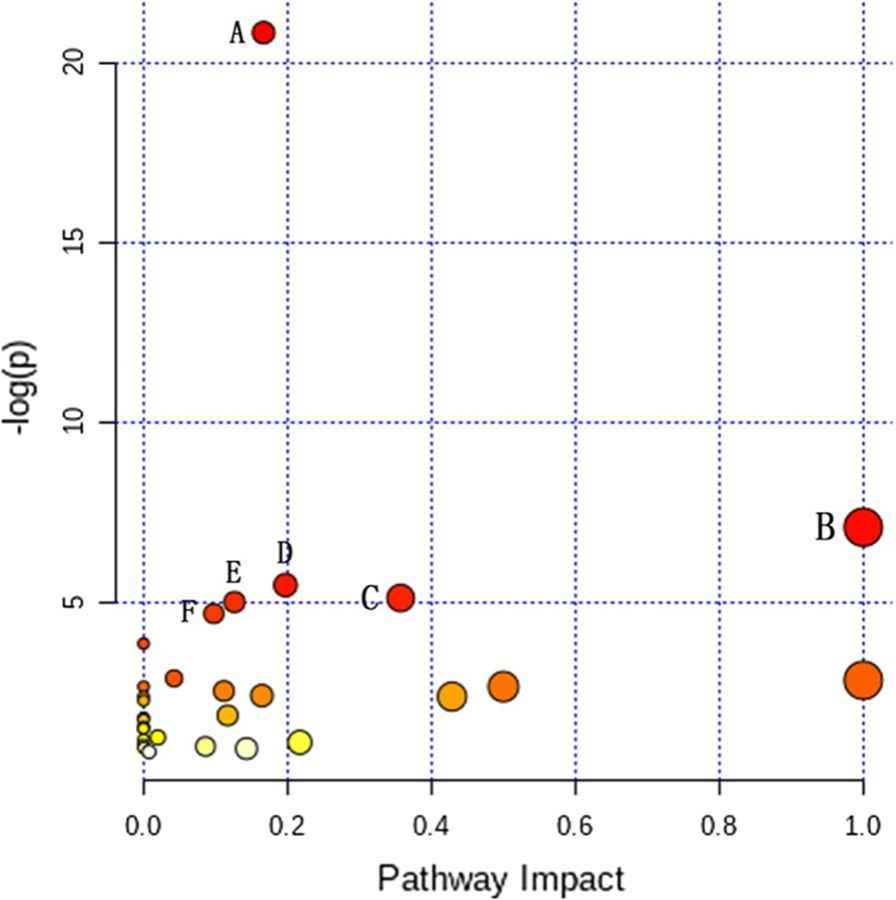
The metabolome view map of relevant metabolic pathways in periapical periodontitis metabolic profiles. The ordinate with *p* value (take the negative common logarithm, that is, −log_10_ *p*) is calculated from the enrichment analysis; the abscissa with an impact value of the pathway is obtained from topology analysis. Each bubbles represents a metabolic pathway of the sample. Regarding the ordinate, the higher the −log_10_ *p* of the bubble is, the smaller the *p* value of the sample is. Regarding the abscissa, the bigger the size of the bubble is, the greater the impact value of the sample is. **A** Aminoacyl-tRNA biosynthetic pathway; **B** Phenylalanine, tyrosine and tryptophan biosynthesis pathway; **C** Phenylalanine metabolism pathway; **D** Alanine, aspartate and glutamate metabolism pathway; **E** Cysteine and methionine metabolism pathway; **F** Amino sugar and nucleotide sugar pathway

The identified biomarkers responsible for periapical periodontitis were further analysed by MetaboAnalyst 4.0 (https://www.metaboanalyst.ca/). The pathway analysis (Fig. 7) showed that the 18 potential biomarkers of periapical lesions were mapped to 26 pathways. Six pathways with an impact value > 0.1 and a *p* value < 0.05 were selected as the significantly different pathways in the comparison between grades I and III and grades II and III. Among the six metabolic pathways, five pathways were associated with amino acid metabolism, including aminoacyl-tRNA biosynthetic, phenylalanine, tyrosine, and tryptophan biosynthesis; phenylalanine metabolism; alanine, aspartate, and glutamate metabolism; and cysteine and methionine metabolism; one pathway was related to sugar metabolism. Moreover, 13 metabolites were correlated with the six metabolic pathways mentioned above (Table 3).

**Table 3:** Metabolite changes in the metabolic pathway analysis of periapical periodontitis

	Metabolic pathway	The total number of metabolites	The total number of differential metabolites	Metabolites	*P* value	**−**Log(*p*) value
1	Aminoacyl-tRNA biosynthesis	48	9	L-Asparagine, L-Phenylalanine, L-Serine, L-Methionine, L-Valine, L-Alanine, L-Tryptophan, L-Tyrosine, L-Glutamine	0.00	21.47
2	Phenylalanine, tyrosine and tryptophan biosynthesis	4	2	L-Phenylalanine, L-Phenylalanine	0.00	7.19
3	Alanine, aspartate and glutamate metabolism	28	3	L-Asparagine, L-Alanine, L-Glutamate	0.00	5.63
4	Phenylalanine metabolism	10	2	L-Phenylalanine, L-Tyrosine	0.01	5.22
5	Cysteine and methionine metabolism	33	3	L-Serine, L-Methionine, 3-methylthiopropionate	0.01	5.16
6	Amino sugar and nucleotide sugar metabolism	37	3	*N*-Acetylmannosamine, *N*-acetyl-D-glucosamine	0.01	4.83

**Fig. 8:**
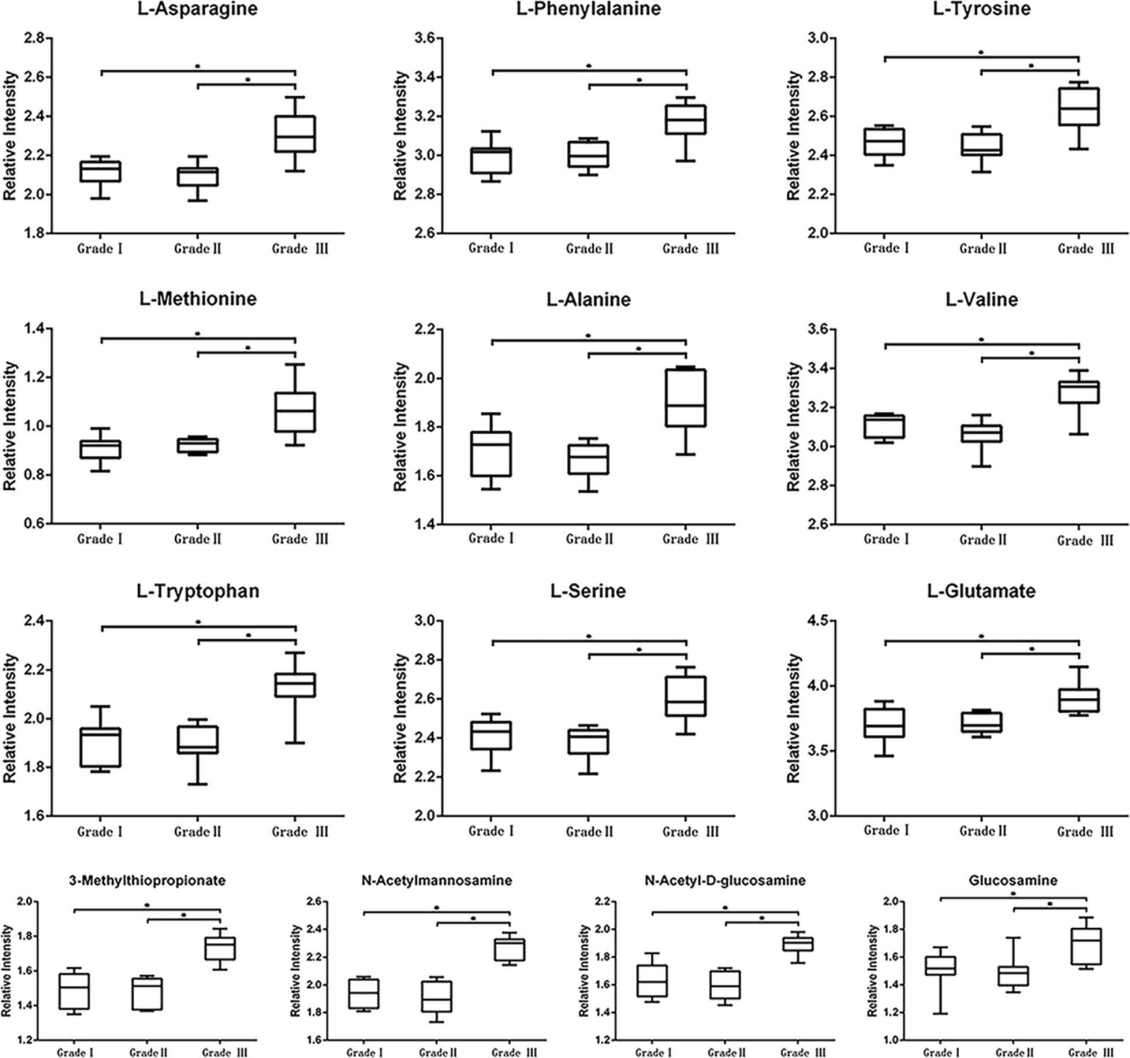
Changes in relative concentrations of 13 key metabolites in different immune cell infiltration levels (*p < 0.05)

Figure 8 shows the relative intensity of 13 key metabolites in the samples of periapical periodontitis with different inflammatory grades. The relative concentrations of 13 metabolites in the grade III periapical periodontitis group were significantly increased compared with those in the grade I and grade II periapical periodontitis group (*p* < 0.05). However, there was no significant difference in the relative concentrations of 13 metabolites between the grade I and II periapical periodontitis groups (*p >* 0.05).

**Fig. 9:**
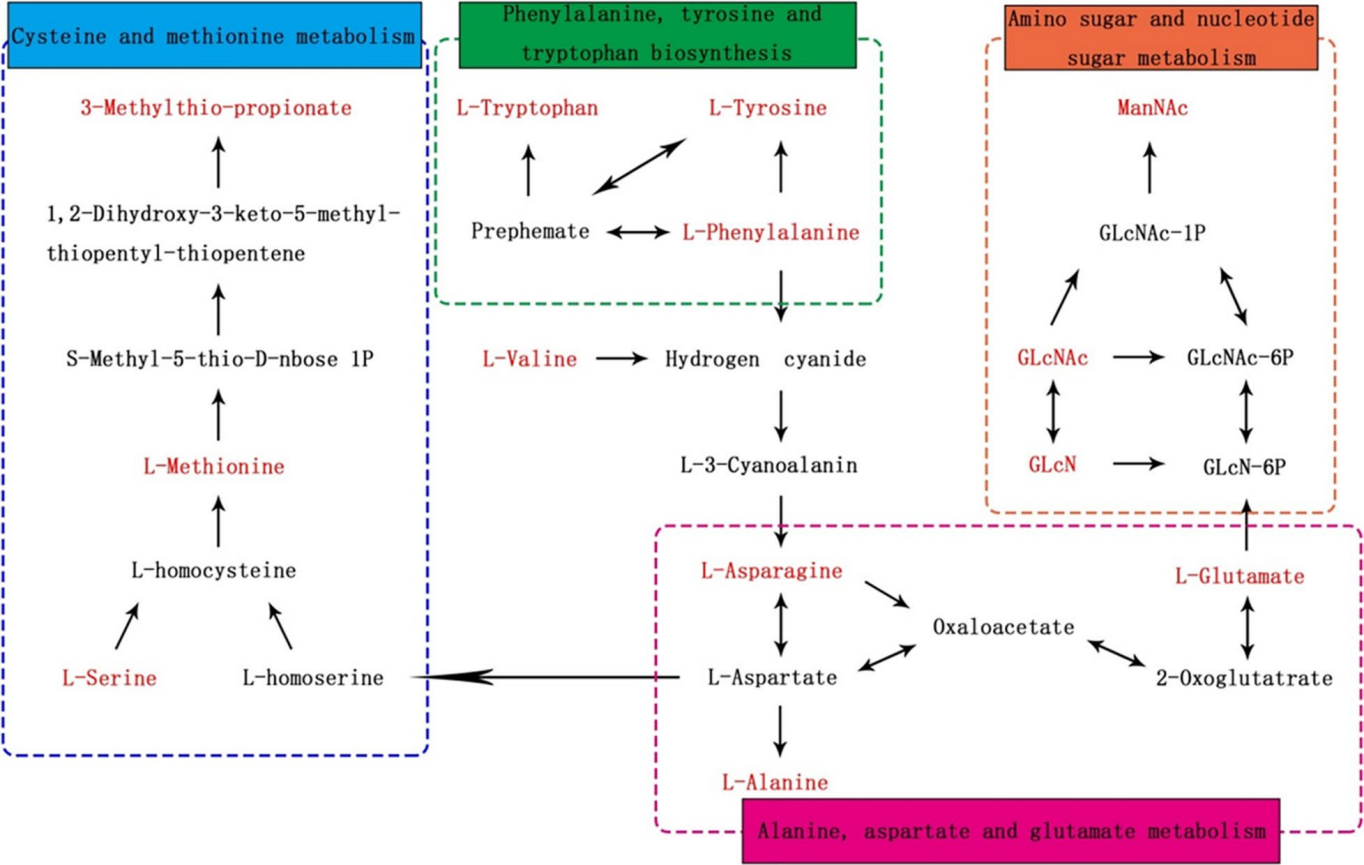
Key biomarkers and the relevant metabolic networks identified from the KEGG database. Red words represent 13 key biomarkers in periapical periodontitis and indicate the increased expression of metabolites around the periapical lesions

The metabolic networks revealed potential connections between the 13 key biomarkers and the relevant metabolic pathways in periapical periodontitis (Fig. 9). Asparagine of the alanine metabolic pathway was potentially regulated by or connected to the phenylalanine, tyrosine and tryptophan biosynthesis pathways; aspartate of the aspartate metabolic pathway was potentially regulated by or connected to the cysteine and tryptophan metabolic pathways; and glutamate of the glutamate metabolic pathway was potentially regulated by or connected to the amino sugar and nucleotide metabolic pathways. Compared to those associated with lower degrees of inflammation, the key biomarkers in these metabolic pathways associated with higher degrees of inflammation were significantly increased in periapical periodontitis (*p < *0.05) (Fig. 9).

### Microbiome analysis of periapical periodontitis

**Fig. 10:**
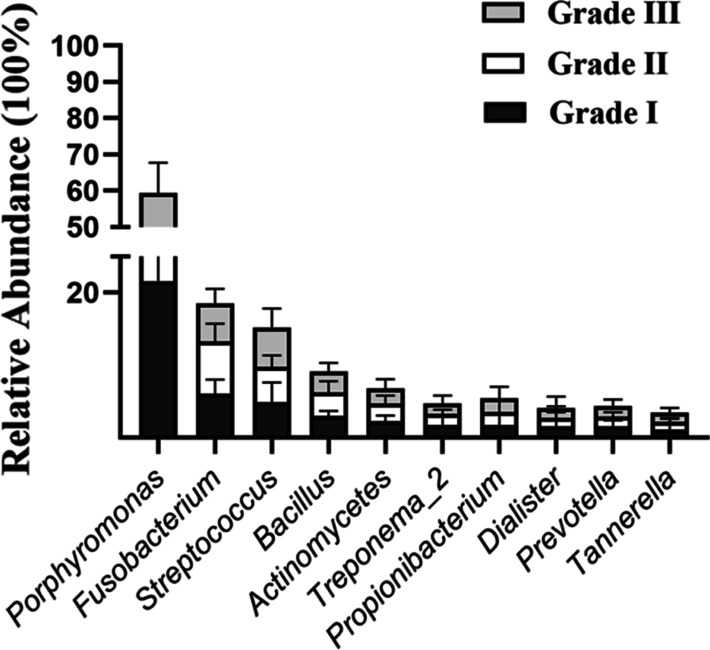
Comparisons of relative abundance of ten major microorganisms from periapical periodontitis with different inflammation degrees. *p* values are based on variance analysis and all *p* >0.05

After importing 16 S rRNA high-throughput sequencing data into QIIME 1.8.0 software, the data showed that the bacterial populations associated with periapical periodontitis were characterized by the presence of *Porphyromonas*, *Fusobacterium*, *Streptococcus*, *Bacillus*, and *Actinomycetes*. Regardless of the severity of inflammation, the proportions of microorganisms, in a sequence from higher to lower, were *Porphyromonas*, *Fusobacterium*, and *Streptococcus*. There was no significant correlation between the inflammatory grade and the type or abundance of infectious microorganisms associated with periapical periodontitis using variance analysis (all *p* >0.05). Representative microorganisms of periapical periodontitis with different inflammatory grades are presented in Fig. 10.

### Correlation between the metabolome and microbiome of periapical periodontitis

**Fig. 11:**
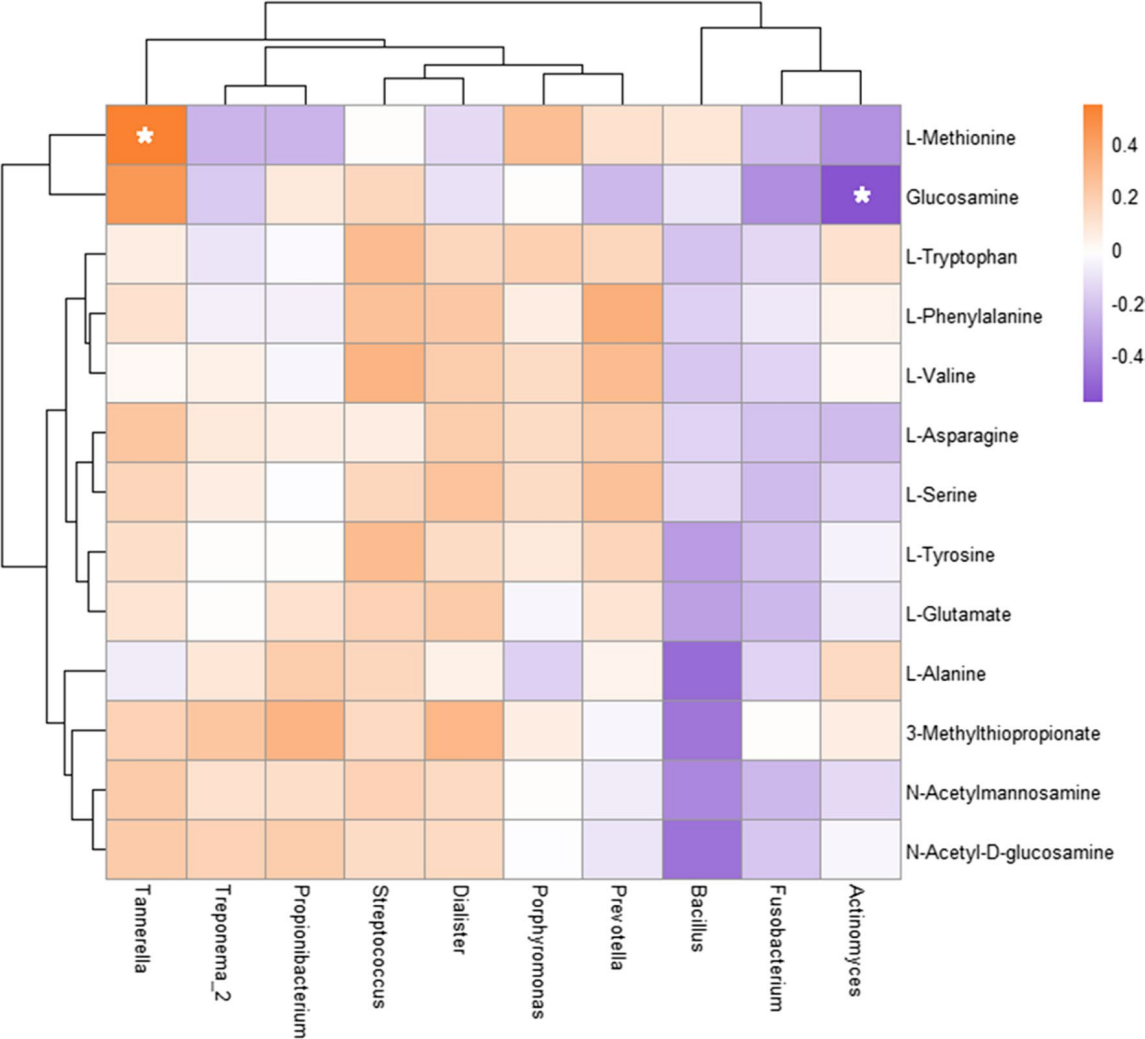
Heatmap of correlation between major metabolites and relative abundance of major microorganisms in periapical periodontitis. Metabolite and microbial data were clustered using Spearman’s correlation analysis. The correlation (R) value is expressed in different colors in the figure. The legend on the right is the color range of different R values. *p < 0.05

The correlation between thirteen key metabolites and the type or relative abundance of ten major microorganisms was further analysed (Fig. 11). The abundance of *Actinomycetes* was negatively correlated with the abundance of glucosamine (GlcN), while the abundance of *Tannerella* was positively correlated with the abundance of L-methionine.

## Discussion

In recent years, metabolome or microbiome analysis techniques have emerged as objective tools for identifying the metabolomic mechanisms underlying the development of chronic inflammatory disorders mainly caused by bacterial infection. A microbiome-metabolome study revealed that the gut microbiota was associated with glycine-conjugated metabolites and polyamine metabolism in chronic kidney disease [26]. Recently, metabolomics of oral diseases has made certain progress. Gokul analysed and identified serum metabolites in oral leukoplakia and oral squamous cell carcinoma using quadrupole time of flight-liquid chromatography-mass spectrometry [27], and Barnes performed an unbiased metabolomic profiling of gingival crevicular fluid (GCF) collected from healthy, gingivitis and periodontitis sites in humans using liquid and gas chromatography mass spectrometry [28]. However, the local microbiome-metabolome alterations that occur in the development of periapical periodontitis have not been discussed in previous studies.

In this study, the levels of phenylalanine, tyrosine, and tryptophan in the phenylalanine metabolism pathway increased significantly in the grade III periapical periodontitis samples compared with the grades I and II periapical periodontitis samples. High levels of phenylalanine inhibit the activity of 5-hydroxytryptophan decarboxylase and decrease the synthesis of 5-hydroxytryptamine (5-HT) from tryptophan [17]. Moreover, tyrosine is a nonessential amino acid that is synthesized from phenylalanine by phenylalanine hydroxylase. This result suggested that the high level of phenylalanine might be positively correlated with the increase in tryptophan and tyrosine levels. A previous study demonstrated that tryptophan has strong anti-inflammatory activity, which might be explained by its significant inhibitory effect on IL-8 secretion by interfering with TNF-α-activated inflammatory signal transduction [23]. In this study, the overproduction of phenylalanine in periapical periodontitis might subsequently increase tryptophan production. Therefore, hyperactivity of tryptophan in periapical periodontitis might decrease the production of local proinflammatory factors and increase the local anti-inflammatory activity to physically protect against bacterial inflammation.

The levels of alanine, glutamine and asparagine in the alanine, aspartate and glutamate metabolic pathways were increased in grade III periapical periodontitis samples compared with the grade I and II periapical periodontitis samples. Alanine and asparagine play central roles in maintaining metabolism, protein synthesis, nitrogen balance and immune responses in cells [20]. Moreover, alanine, glutamine and asparagine have anti-inflammatory activities that are related to TNF-α and IL-1β, which can activate osteoclasts and stimulate bone resorption [18]. Therefore, the increase in alanine, glutamine and asparagine levels in periapical periodontitis might suppress the production of proinflammatory cytokines and the expansion of inflammation in periapical periodontitis.

The levels of methionine, serine, and 3-methylthiopropionate in the cysteine and methionine metabolic pathways were increased obviously in the grade III periapical periodontitis samples compared with the grade I and II periapical periodontitis samples. Methionine has important antioxidant and antiresorptive effects by inhibiting the propensity of mononuclear cells to develop into functional osteoclasts. 3-Methylthiopropionate is an intermediate in the methionine catabolism pathway [15]. Therefore, elevated levels of methionine and 3-methylthiopropionate might regulate the local production of proinflammatory cytokines, suppress the differentiation of osteoclasts, and attenuate bone loss in periapical periodontitis. In addition, serine is an essential metabolite for regulating clonal T lymphocyte expansion in adaptive immunity [21]. Therefore, the increased expression of serine in periapical periodontitis could be associated with autoimmune regulation in periapical periodontitis.

The levels of glucosamine (GlcN), N-acetylmannosamine (ManNAc), and N-acetyl-D-glucosamine (GlcNAc) in the cysteine and methionine metabolic pathways were increased significantly in the grade III periapical periodontitis samples compared with the grade I and II periapical periodontitis samples. ManNAc is the metabolite of GlcN and GlcNAc. Both GlcN and GlcNAc attenuate IL-1β, IL-6, and TNF-α expression in normal human articular chondrocytes, and GlcN plays a novel role in the alleviation of oxidative stress and lung inflammation in rats [14]. These findings indicate that GlcN and GlcNAc might directly reduce local inflammation in periapical periodontitis by inhibiting anti-inflammatory cytokine production and alleviating oxidative stress.

The microbial community associated with in periapical periodontitis is complex and diverse. In this study, the severity of inflammation had no obvious correlation with the abundance of single microorganisms, suggesting that persistent periapical periodontitis is related to combined polymicrobial infections. Gomes et al. investigated the presence of nine bacterial species in root-filled teeth associated with periapical lesions using the same method and found that *Enterococcus faecalis, Porphyromonas gingivalis* and *Peptostreptococcus micros* were the most frequently identified species [1]. Federico Mussano et al. found that facultative anaerobes were dominant in periapical granulomas and radicular cysts, but the anaerobic types were the most abundant in radicular cyst samples [29]. In this study, *Porphyromonas, Fusobacterium*, and *Streptococcus* were the predominant species in all three groups of periapical periodontitis samples, regardless of the severity of inflammation. The difference in the microbial species may be attributed to the difference in human ethnicities, individual immune systems, clinical materials, tissue location, sample size and treatment procedures [30].

In this study, the abundance of *Actinomycetes* was negatively correlated with the abundance of glucosamine (GlcN). Studies show that GlcN has immune regulatory functions and regulates the activation of IL-1β [14]. *Actinomyces* has been commonly found in primary root canal infections and is also known as an important microorganism in the development of persistent apical periodontitis lesions [31]. Further study showed that GlcN is positively related to amino sugar metabolism in *Actinomycetes*, and it functions by enhancing the role of glycogen and improving the catalytic performance. In addition, the abundance of *Tannerella* was positively correlated with that of L-methionine. L-Methionine inhibits the transdifferentiation of osteoclasts and the synthesis of proinflammatory cytokines, such as IL-1α [15]. *Tannerella* is one of the prevalent microorganisms in both primary periapical periodontitis and endodontic failure and can induce the activation of inflammasomes in periapical periodontitis [32]. *Tannerella* might increase the inflammatory grade by inhibiting the metabolism of L-methionine in periapical periodontitis; however, there are still no studies on the correlation between *Tannerella* and L-methionine, which needs to be discussed in the future.

Here, we investigated the relationship among local metabolome alterations, inflammatory grades, and types and abundances of microorganisms associated with periapical periodontitis. This study found that periapical periodontitis was related to changes in amino acid, sugar and fatty acid metabolism, and the more severe the inflammation was, the more obvious the metabolic changes were. This finding indicates that the local immune system and metabolism are involved in the occurrence and development of periapical periodontitis. Additionally, metabolites have strong anti-inflammatory activity and can inhibit the production of osteoclasts. The increased levels of these metabolites suggest that physiological regulation occurs during the development of periapical periodontitis. The mechanisms underlying this observation need to be further studied.

## Conclusion

The study revealed that inflammatory grades are related to local metabolic changes in periapical periodontitis. The local changes in the metabolites GlcN and L-methionine were correlated with the changes in the abundance of the major microorganisms *Actinomycetes* and *Tannerella*, respectively. The findings provide important insights into local metabolome regulation during the progression of periapical periodontitis. However, further investigation is still required to explore the molecular interaction mechanisms underlying this issue.

## Data Availability

The datasets used and analysed during the current study are available from the corresponding author on reasonable request.

## Abbreviations

PCA: Principal components analysis
OPLS-DA: Orthogonal partial least squares discriminant analysis
LC–MS: UHPLC/QTOF–MS
QC: Quality control
VIP: Variable importance in project
GlcN: Glucosamine
ManNAc: *N*-Acetylmannosamine
GlcNAc: *N*-acetyl-D-glucosamine
